# The Use of AI-Powered Thermography to Detect Early Plantar Thermal Abnormalities in Patients With Diabetes: Cross-Sectional Observational Study

**DOI:** 10.2196/65209

**Published:** 2025-06-13

**Authors:** Meshari F Alwashmi, Mustafa Alghali, AlAnoud AlMogbel, Abdullah Abdulaziz Alwabel, Abdulaziz S Alhomod, Ibrahim Almaghlouth, Mohamad-Hani Temsah, Amr Jamal

**Affiliations:** 1 Amplifai Health, 3053 Yaqoub al kandi, Al Nafel 7762, Riyadh, 13312, Saudi Arabia, 966 569272937; 2 College of Medicine, King Saud University, Saudi Arabia; 3 Seha Virtual Hospital, Ministry of Health, Saudi Arabia; 4 Health and Wellbeing Sector at Neom, Neom, Saudi Arabia

**Keywords:** AI, computer-assisted, digital health, eHealth, diabetic foot, diabetes, monitoring, detect, detection, diagnosis, diagnostic, thermography, thermology, thermal, population health, artificial intelligence, foot ulcer, diabetic, image, imaging

## Abstract

**Background:**

Diabetic foot problems are among the most debilitating complications of diabetes mellitus. Diabetes prevalence and complications, notably diabetic foot ulcers (DFUs), continue to rise, challenging health care despite advancements in medicine. Traditional DFU detection methods face scalability issues due to inefficiencies in time and practical application, leading to high recurrence and amputation rates alongside substantial health care costs. Human medical thermography could significantly enhance disease monitoring and detection, including DFUs.

**Objective:**

This study evaluated the efficacy of artificial intelligence–powered thermography in detecting plantar thermal patterns that differentiate between adult patients with diabetes with no visible foot ulcers and healthy individuals without diabetes.

**Methods:**

This cross-sectional observational study included 200 patients—100 healthy and 100 with diabetes without a visible foot ulcer. Initial data were gathered through a questionnaire. Participants were prepared for thermal imaging to capture plantar thermal patterns. All collected data, including thermal images and questionnaire responses, were stored on a password-protected computer to ensure confidentiality and data integrity.

**Results:**

In this study, participants were categorized into 2 groups: a healthy control group (n=98) with no prior diabetes or peripheral artery disease diagnosis and normal circulatory findings, and a group with diabetes (n=98) comprising patients with diabetes, regardless of peripheral circulatory status. Temperature analysis indicated a wider range in the group with diabetes (18.1-35.6 °C) than in the healthy controls (21.1-35.7 °C), with the former showing significantly higher mean temperatures (mean 29.0 °C, SD 3.0 °C) than controls (mean 28.9 °C, SD 2.8 °C; *P*<.001). Analysis of both feet revealed significantly greater differences between feet in the group with diabetes and the controls (control: mean 0.47 °C, SD 0.43 °C; group with diabetes: mean 1.78 °C, SD 1.58 °C; *P*<.001; 95% CI 0.99-1.63). These results identified clinically relevant abnormalities in 10% of the cohort with diabetes, whereas no such findings were observed in the control group. We used a linear regression model to indicate that being diagnosed with diabetes is a significant predictor of abnormal temperature, while age and sex were not found to be significant predictors in this model.

**Conclusions:**

DFUs pose a significant health risk for patients with diabetes, making early detection crucial. This study highlights the potential of an artificial intelligence–powered computer vision system in identifying early signs of diabetic foot complications by differentiating thermal patterns between patients with diabetes with no visible ulcers and healthy individuals. The findings suggest that the technology could improve early diagnosis and outcomes in diabetic foot care, although further research is needed to fully validate its effectiveness. The ability of the technology to detect compromised blood supply indicates its value in preventative clinical strategies.

## Introduction

### Diabetic Foot Ulcers

Diabetes affects 1 in 10 adults worldwide (537 million) [1]. This number is predicted to rise to 643 million by 2030 and 783 million by 2045. In the Middle East and North Africa region, the prevalence is higher as it affects 1 in 6 adults (73 million) [1]. Despite advances in medical therapies, the prevalence of diabetes mellitus and diabetes-related complications continues to rise [2].

Diabetic foot problems are among the most debilitating complications of diabetes mellitus. It is commonly referred to as diabetic foot ulcer (DFU). The International Working Group on the Diabetic Foot defines a DFU as a break of the skin of the foot that includes minimally the epidermis and part of the dermis among patients with diabetes mellitus [3]. It is estimated that one-third of people with diabetes will develop a DFU during their lifetime [4]. Armstrong et al [5] emphasized the alarming statistic that every 20 seconds, a lower limb is amputated due to complications of diabetes, with 85% of these amputations preceded by a foot ulcer. The mortality risk at 5 years for individuals with DFUs is 2.5 times higher than for those with no ulcers [4]. Furthermore, Driver et al [6] revealed that DFUs and lower extremity amputations are not only markers of poor health but also independent risk factors associated with premature death. Unfortunately, even after a DFU has been resolved, recurrence is common and is estimated to be 40% within 1 year, 60% within 3 years, and 65% within 5 years [4].

### Economic Burden

Diabetes foot care costs are the single largest category of diabetes-related medical costs. Kurkela et al [7] found that the cost of care for patients with a foot ulcer is 5.4 times higher than that for patients with diabetes with no ulcers, accentuating the heightened financial burden linked to DFUs. The study by Driver et al [6] stated that about one-third of the direct costs of diabetes is attributable to care for diabetic foot disease. Leveraging thermography as a screening modality for DFUs has already demonstrated a noteworthy cost-saving potential, as evidenced by the findings of Everett and Mathioudakis [8]. Despite the requirement for human thermographers in their methodology, their study projected substantial savings through the integration of thermography as a standard procedure.

### Current Challenges in Early Detection of DFUs

Unfortunately, DFUs can be difficult to detect, especially in the early stages when it is not visible to the human eye. This is due to a number of factors, including inconsistent screening guidelines, limited awareness among patients and providers, and the frequent occurrence of silent or nonstandard symptoms [4,9,10]. To improve the early detection of DFUs, it is important to develop and implement universal screening guidelines, increase awareness among patients and providers, and develop and implement better screening tools and methods.

### Role of Thermography in DFU Detection

**Figure 1.**
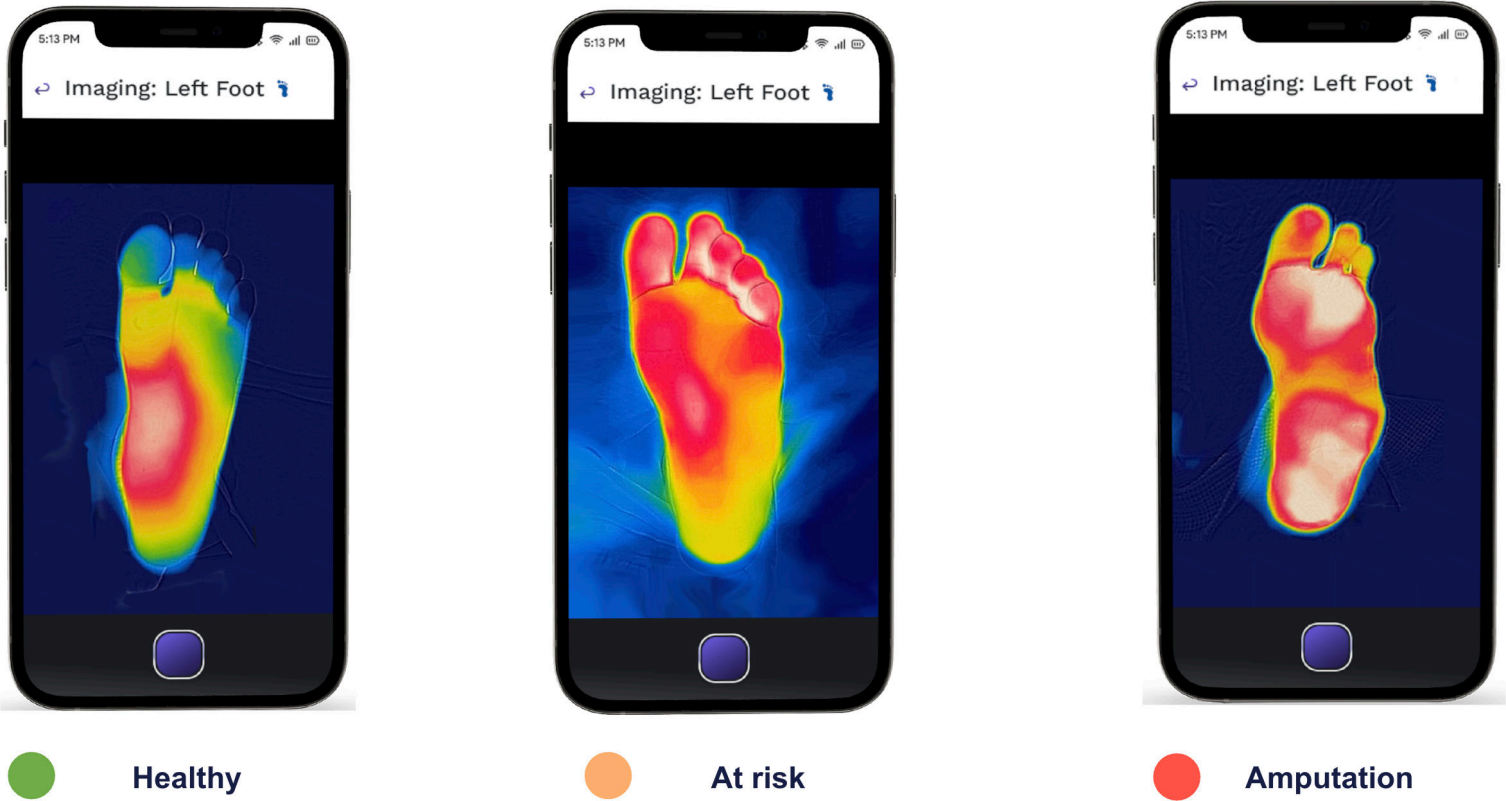
Typical thermal images of plantar region for healthy patients, at-risk patients, and patients who underwent amputation.

Medical thermography results from decades of research and development in the performance of infrared imaging equipment, standardization of technique, and clinical protocols for thermal imaging [11,12]. It could visualize diseases not readily detected or monitored by other methods. It is a fast, passive, noncontact, and noninvasive imaging method that has been used by numerous peer-reviewed studies [13]. There is an increase in publications and high-impact studies, emphasizing thermography’s importance as a crucial tool for the early detection, prevention, and management of diabetic foot issues [14]. The American Academy of Thermology (AAT) established guidelines for the use of thermography in the evaluation of patients with diabetes. These guidelines provide recommendations for the use of thermal imaging in the detection and monitoring of diabetic neuropathy, including protocols for image acquisition and interpretation [15]. Figure 1 shows the thermal images of the lower extremities of patients with diabetes.

A major limitation of the current state of medical thermography is that even the most skilled human thermographer can only observe, analyze, and successfully interpret a limited number of thermograms. Computers, however, can process an image efficiently and extract useful information. Leveraging artificial intelligence (AI) algorithms, specifically, computer vision, can objectively observe the findings and minimize interobserver variability.

Thermography is helpful for the early detection of abnormalities of the foot by analyzing asymmetries and local temperature changes over time. Assessing temperature differences can enable the early detection of ulcers [16,17]. The application of thermal imaging for the detection of diabetic foot complications is based on the premise that variations in plantar temperature are associated with these types of complications [17-24]. Several papers highlighted that high temperature gradients between feet may predict the onset of foot ulcers [25-28]. Furthermore, the international working group of diabetic foot recommends a person with diabetes who is at moderate or high risk of foot ulceration to self-monitor foot skin temperatures once per day to identify any early signs of foot inflammation and help prevent a foot ulcer [29].

The rapid development of handheld smartphone-based thermal infrared imagers presents a creative solution for detecting and monitoring DFUs [30]. To address the lack of thermographers, practical AI algorithms are needed to automate the process of image acquisition and analysis. These rapidly expanding, low-cost, and widely available resources can help predict one’s risk of developing foot ulcers, potentially saving limbs and lives. In this study, we will leverage AI technologies that are deployed on a smartphone-based thermal imager and application.

### Rationale for the Study

The purpose of this study is to evaluate the efficacy of AI- powered thermography in detecting plantar thermal patterns that differentiate between adult patients with diabetes with no visible foot ulcers and healthy individuals with no diabetes.

## Methods

### Study Design

This is a cross-sectional observational study. The data collection phase spanned from June 2023 to February 2024. This time frame was sufficient to recruit people into the healthy group and individuals with no diabetes group.

### Study Location

Participants were recruited from King Khalid University Hospital, part of the King Saud University Medical City, a multidisciplinary facility offering general and subspecialty medical services, including primary, secondary, and tertiary care. Patients with diabetes were consecutively recruited from the diabetes clinics during the study period, reflecting real-world clinical practice. Healthy participants, on the other hand, were recruited from those visiting the hospital for routine check-ups or preventive care during the same period. All eligible individuals meeting the inclusion criteria were invited to participate, ensuring representativeness of the control group. A research assistant (RA) was present to facilitate recruitment and collect the necessary data. This consecutive sampling approach ensured that all eligible patients attending the clinic were considered for participation.

### Inclusion Criteria

The study included participants who were older than 18 years; healthy individuals, with no history of diabetes or cardiovascular disease (n=100); and have been diagnosed with diabetes (n=100). The inclusion of healthy participants as controls allowed for clear differentiation between diabetic and nondiabetic thermal patterns, emphasizing the distinct thermal asymmetry observed in patients with diabetes.

### Exclusion Criteria

Eligibility for participation was assessed through self-report during recruitment. The study excluded participants who have a visible foot pathology, such as visible ulcers, infections, or amputations, and are unable to stand without assistance due to the higher risk of falling or injuring themselves during the study. Participants who meet the inclusion criteria and are willing to participate were asked to provide their informed consent.

### Withdrawal of Participants From the Assessment

Participants were free to withdraw from the study at any time without giving a reason. Patients were advised that if they requested to withdraw from the study, at any time during the trial, then this would have no negative consequences.

### Description of the Technology

**Figure 2.**
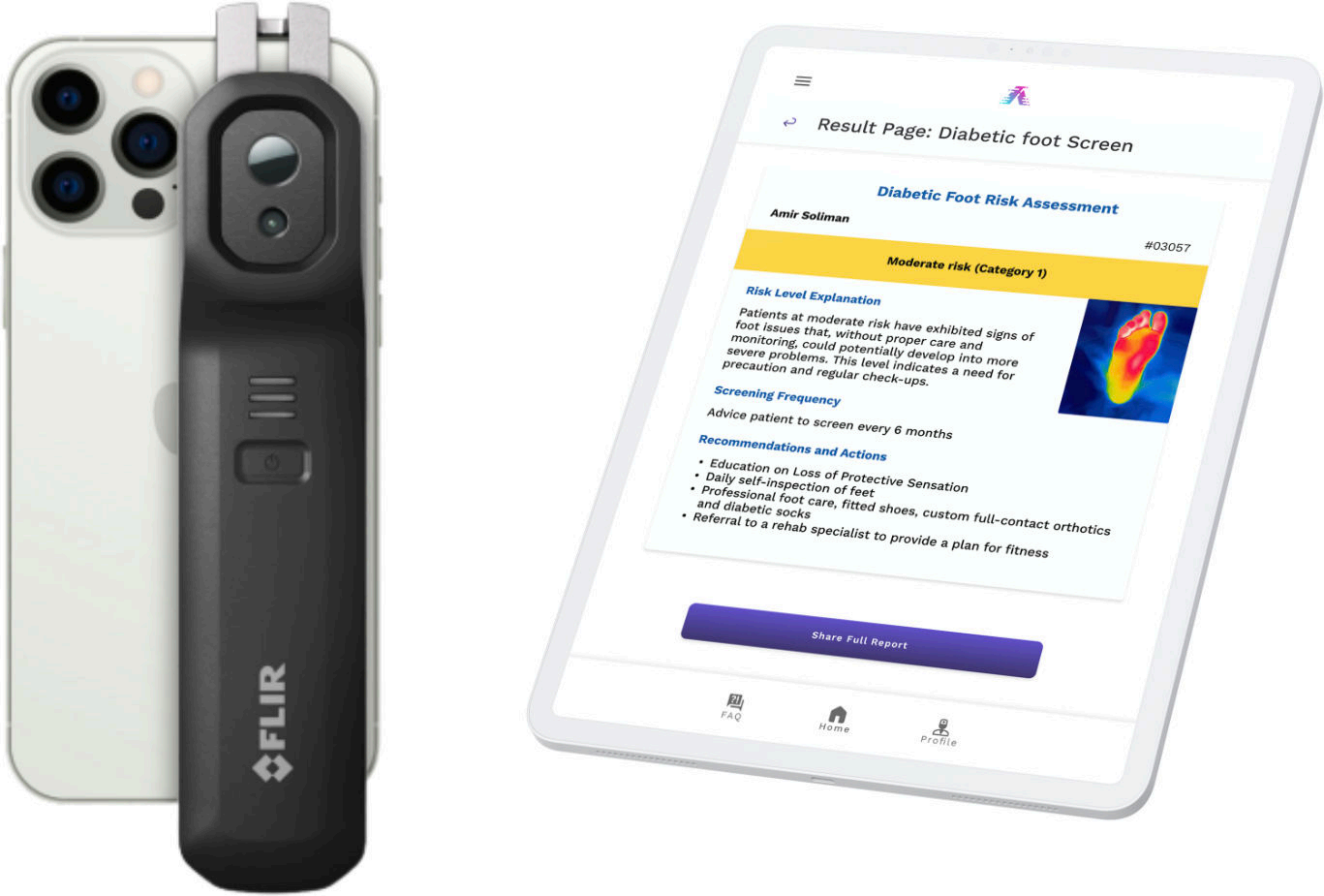
Thermal imaging device.

We have created a noninvasive system to identify diabetic foot complications at an early stage. We leverage off-the-shelf thermal cameras, compatible with smartphones or tablets, to capture detailed thermal images of participants’ feet (Figure 2). We also used AI-based algorithms to perform semantic segmentation and reduce sensor noise in the captured thermograms. The AI models were trained to extract the plantar region as the region of interest and suppress any background or sensor noise. In addition, the AI was able to detect asymmetric thermal emission of 2.2 °C or greater, which can be indicative of pathology in a properly cooled participant [3,15,26,28,31,32].

The system used AI to process images efficiently and extract useful information. It made the findings more objective and minimized interobserver variability. This could result in faster throughput and through a centralized cloud-based processing where samples were anonymized by removing identifiable information from the data., increasing thermographic accuracy and reliability.

The technology analyzes thermal images captured from specific thermal camera models, the FLIR ONE Edge Pro. We selected the FLIR One Edge for its practicality and ease of use in clinical settings. While its resolution is lower than the AAT recommendation of 320×240 pixels, several studies have successfully used the FLIR One, which has an even lower resolution of 80×60 pixels, and concluded that it can effectively capture thermal signals indicative of DFUs [31,33,34]. The software identifies temperature variations consistent with inflammation and ulcerative patterns, signaling potential DFUs. The system records, stores, and transmits usage events from thermal cameras to a remote storage system.

### Data Collection

Before enrolling participants, the RA was trained to follow the study protocol, including participant recruitment, questionnaire administration, and thermal image capture. The RA completed a questionnaire via an app that included questions about the participant’s age, sex, type of diabetes, duration of diabetes (years), hemoglobin A_1c_ (HbA_1c_), BMI, physical activity habits, smoking habits, and history of hypertension. In addition, they completed Inlow’s 60-second Diabetic Foot Screen to assess the foot [35]. After completing the questionnaire, the RA prepared the participant and completed the thermal imaging.

### Experimental Equipment and Procedure

The results of infrared thermography can be influenced by various environmental, individual, and technical factors that affect human skin. To obtain accurate results, the thermal images of the participants in the study were carried out in compliance with the protocols and guidelines set by the AAT [15].

To maintain a stable blood flow to the feet, we asked participants to remove their shoes and socks and sit with their legs hanging freely for at least 10 minutes before the measurements were made. We maintained the humidity in the room to ensure that there is no moisture buildup on the skin, perspiration, or vapor levels that can interact with radiant infrared energy. Relative humidity below 70% is generally acceptable. The temperature was maintained between 19 ºC and 25 ºC. The camera was set about 1 m from the foot (the region of interest will fill ~75% of the image), and thermal masking was used to ensure a homogeneous background. Images of the plantar aspect of the feet were recorded for analysis.

### Ethical Considerations

Ethical approval for this study was obtained from King Saud University institutional review board (E-23‐7866). Before agreeing to participate, all participants were informed about the nature of the research project, possible risks and benefits, and their rights as research participants. All participants completed a written consent form. They were also given a copy of the consent form. No compensation was provided to the participants. This decision was communicated to participants during the consent process, ensuring complete transparency.

Participants were coded with a specific clinical investigation identification number. All participants were registered in a participant identification list (participant enrollment and identification list) that connects the participant’s name and personal number with a clinical investigation identification number. All data were registered, managed, and stored in a manner that enables correct reporting, interpretation, and verification.

### Statistical Analysis

A database of the questionnaire results was created using unique nonidentifying numbers. The information is password-protected. Before conducting the analysis, data were cleaned and coded using Python Pandas NumPy and SciPy packages (pandas). Each item was discussed, and a decision concerning its eligibility and entry was made. The characteristics of participants are summarized with percentages for categorical variables and mean (SD) values for continuous variables. To compare the mean ages and BMI values between the healthy group and the group with diabetes, independent 2-sample 2-tailed *t* tests were conducted. For categorical variables, such as sex and health conditions, we used 2-proportion *z* tests to compare both groups.

For the correlation analysis, the foot skin temperature obtained is the average for the entire plantar region. We used the independent 2-tailed *t* test and the effect size (Cohen *d*) to compare foot skin temperature in healthy participants versus participants with diabetes. The level of significance is set at *P*<.05.

In addition, we conducted an ordinary least squares (OLS) regression to examine the relationship between temperature delta (difference in plantar temperatures between feet) and several predictor variables. The primary predictor of interest was diabetes status (healthy vs diabetic). To control for potential confounders, the model also included age, sex, BMI, physical activity level, diabetes duration, diabetes type, HbA_1c_, and the presence of retinopathy. The OLS regression analysis was conducted to determine the significance and strength of the predictors in explaining the variance in the temperature delta. Statistical significance was set at *P*<.05.

While we did not perform a formal sample size calculation prior to the study, our sample size of 200 participants (100 individuals with diabetes and 100 healthy controls) was determined based on practical considerations, including the availability of eligible participants during the study period and the resources at our disposal. We aimed to ensure that the sample was sufficiently large to capture variability in thermal patterns between the 2 groups and to allow for meaningful statistical analyses. To support the adequacy of our sample size, we reviewed several related studies [17,19,21,24], many of which used smaller sample sizes while investigating similar research questions.

## Results

### Participant Characteristics

Participants were divided into 2 groups. Individuals with normal circulatory findings and with no earlier diagnosis of diabetes or peripheral artery disease (PAD) were assigned to the healthy control group (n=98). All patients with diabetes, with or with no peripheral circulatory disturbance, were assigned to the group with diabetes (n=98). It is important to note that the group with diabetes did not have a visible foot ulcer. Approximately 61% (119/196) of participants were female, with a mean age of 39.2 (SD 15.5) years. The average BMI was 26.4 (SD 5.6) kg/m². There was a significant difference between both groups and the group with diabetes was slightly older and had a higher BMI. Patients with diabetes exhibited various comorbidities: cardiovascular disease (16.9%, 33/98), retinopathy (15.4%, 30/98), neuropathy (11.8%, 23/98), and peripheral vascular disease (12.8%, 25/98). Furthermore, 15.9% of patients with diabetes reported poor glycemic control. The group with diabetes included 57.14% (56/98) with type 1 diabetes and 41.84% (41/98) with type 2 diabetes, with a mean diabetes duration of 15.38 (SD 8.99) years. The mean HbA_1c_ level was 7.37%, with an SD of 1.56%. Table 1 illustrates the participant characteristics.

**Table 1.** Participant characteristics (N=196).

Variables	Total (N=196)	Healthy group (n=98)	Diabetic group (n=98)	*P* value	95% CI
Age (years), mean (SD)	39.3 (15.5)	33.2 (11.2)	45.7 (16.8)	<.05	8.4 to 16.5
<25	21.7 (1.7)	22.05 (1.3)	21.1 (2.1)	>.05	−2.1 to 0.3
40	31.2 (4.8)	30.7 (4.7)	32.6 (4.6)	>.05	−0.3 to 4.3
>40	55.4 (10.3)	51.1 (7.6)	57 (10.7)	<.05	0.8 to 11.1
BMI (kg/m²), mean (SD)	26.4 (5.6)	24.31 (4.1)	28.75 (6.2)	<.05	2.9 to 5.9
Males, n (%)	76 (38.7)	38 (38.7)	38 (38.7)	>.05	−13.7 to 13.7
Females, n (%)	120 (61.2)	60 (61.2)	60 (61.2)	>.05	−13.7 to 13.7
Cardiovascular disease, n (%)	33 (16.8)	0 (0)	33 (33.6)	<.05	−44.2 to −23.1
Retinopathy, n (%)	30 (15.3)	0 (0)	30 (30.6)	<.05	−40.7 to −20.4
Neuropathy, n (%)	23 (11.7)	0 (0)	23 (23.4)	<.05	−32.5 to −14.4
Peripheral vascular disease, n (%)	25 (12.7)	0 (0)	25 (25.5)	<.05	−34.9 to −16.1
Diabetes type, n (%)					
Type 1	56 (57.14)	N/A^a^	N/A	N/A	N/A
Type 2	41 (41.84)	N/A	N/A	N/A	N/A
Other	1 (1.02)	N/A	N/A	N/A	N/A
Duration of diabetes, mean (SD)	15.38 (8.99)	N/A	N/A	N/A	N/A
HbA_1c_^b^, mean (SD)	7.37 (1.56)	N/A	N/A	N/A	N/A
Physical activity, n (%)					
Never	63 (32.14)	39 (19.9)	24 (12.24)	<.05	0.02 to 0.28
Several times a week	21 (10.71)	7 (3.57)	14 (7.14)	>.05	−0.15 to 0.01
Rarely	58 (29.59)	30 (15.31)	28 (14.29)	>.05	−0.10 to 0.14
Once a week	14 (7.14)	9 (4.59)	5 (2.55)	>.05	−0.03 to 0.11
Daily	40 (20.41)	13 (6.63)	27 (13.78)	<.05	−0.25 to −0.03

a N/A: not applicable.

b HbA_1c_: hemoglobin A_1c_.

### Thermographic Analysis

The temperatures spanned a wider range in the group with diabetes than in the healthy control group, with a range of 18.1-35.6 °C in the group with diabetes and of 21.1-35.7 °C in the control group. The mean temperatures were significantly higher in the group with diabetes than in the control group (*P*<.001). Considering both feet as 1 bloc, the mean temperatures were 28.9 °C (SD 2.8 °C) among controls, and 29.0 °C (SD 3.0 °C; *P*<.001) in the group with diabetes.

Side-to-side comparisons of temperatures revealed significant differences between feet (*P*<.05) at all measurement sites (Table 2). Analysis of both feet revealed significantly greater differences between feet in the group with diabetes compared with controls (control: mean 0.47 °C, SD 0.43 °C vs group with diabetes: mean 1.78 °C, SD 1.58 °C; *P*<.001; 95% CI 0.99-1.63). These results identified clinically relevant abnormalities in 10% of the cohort with diabetes, whereas no such findings were observed in the control group.

**Table 2.** Absolute values of the between-foot temperature differences in healthy control participants and participants with diabetes.

Region	Total (N=196)	Healthy group (n=98)	Diabetic group (n=98)	*P* value	95% CI
Lateral calcaneal artery, mean (SD)	1.182 (1.37)	0.496 (0.44)	1.87 (1.62)	<.05	1.04-1.71
Medial calcaneal artery, mean (SD)	1.197 (1.29)	0.57 (0.47)	1.81 (1.53)	<.05	0.92-1.56
Lateral plantar artery, mean (SD)	1.169 (1.30)	0.54 (0.49)	1.79 (1.54)	<.05	0.92-1.57
Medial plantar artery, mean (SD)	1.128 (1.29)	0.49 (0.45)	1.76 (1.52)	<.05	0.96-1.60
Entire plantar region, mean (SD)	1.13 (1.33)	0.47 (0.43)	1.78 (1.58)	<.005	0.99-1.63

### Regression Analysis of Thermographic Predictors

This analysis examines the relationship between the temperature delta (dependent variable) and the predictor variables (age, sex, BMI, diabetes duration, diabetes type, HbA_1c_, and physical activity, and whether the participant was healthy or diagnosed with diabetes). The results of the OLS regression analysis are summarized in Table 3.

The overall model was not statistically significant (*F*-statistic=1.010; *P*=.46), indicating that the predictors collectively explained only a small proportion of the variance in the temperature delta. The *R*^2^ (uncentered) value was 0.224, and the adjusted *R*^2^ (uncentered) was 0.002, suggesting a weak fit of the model to the data.

However, diabetes status remained a statistically significant predictor of temperature delta (β=5.544; *P*=.02; 95% CI of 1.064-10.024). This confirms that participants with diabetes had significantly greater foot temperature asymmetry than healthy participants, even after adjusting for potential confounders. Retinopathy also emerged as a statistically significant predictor (β=1.3676; *P*=.04; 95% CI of 0.075-2.660), indicating that participants with retinopathy exhibited greater temperature asymmetry than those with no retinopathy. This finding highlights a potential link between microvascular complications and thermal abnormalities, consistent with the established connection between diabetic retinopathy and peripheral microvascular dysfunction.

In contrast, age (β=−.0165; *P*=.45), sex (β=−.5768; *P*=.29), BMI (β=.0366; *P*=.38), physical activity (β=−.1464; *P*=.37), diabetes duration (β=−.0440; *P*=.22), diabetes type (β=−.0264; *P*=.97), and HbA_1c_ (β=−.1430; *P*=.38) were not statistically significant predictors of temperature delta in this model.

These results suggest that diabetes status itself is the strongest predictor of thermal asymmetry, while retinopathy provides an additional clinically meaningful signal. These findings suggest that plantar thermography may capture microvascular abnormalities relevant to broader diabetic complications.

**Table 3.** Regression analysis of predictors for between-foot temperature differences.

Predictor	β coefficient	SE value	*t* test (*df*)	*P* value	95% CI
Intercept	—^a^	—	—	—	—
Age (years)	−0.0165	0.022	−0.756 (42)	.454	−0.060 to 0.028
Diagnosed with diabetes	5.544	2.220	2.498 (42)	.017	1.064 to 10.024
Gender	−0.5768	0.532	−1.084 (42)	.285	−1.651 to 0.497
BMI	0.0366	0.041	0.897 (42)	.375	−0.119 to 0.046
Physical activity	−0.1464	0.161	−0.911 (42)	.367	−0.471 to 0.178
Diabetes duration	−0.0440	0.035	−1.253 (42)	.217	−0.115 to 0.027
Diabetes type	−0.0264	0.611	−0.043 (42)	.966	−1.259 to 1.206
HbA_1c_	−0.1430	0.162	−0.884 (42)	.382	−0.470 to 0.184
Retinopathy	1.3676	0.641	2.135 (42)	.039	0.075 to 2.660

a Not available.

## Discussion

### Principal Findings

This study has indicated that the technology can objectively detect an abnormal thermal pattern in adult patients with diabetes with no visible foot ulcers when compared with healthy individuals with no diabetes. This abnormal heat signature could indicate the presence of a DFU. The skin temperature was significantly different between participants with diabetes and the healthy control group, and the blood skin surface temperature of patients with diabetes was higher than that of the healthy control group. In addition, the technology was able to reveal differences between angiosome areas, as outlined in Table 2. None of the healthy individuals exhibited a temperature delta of 2.2 °C or greater, whereas a subset of patients with diabetes did, highlighting the distinct thermal patterns associated with diabetes.

In addition to diabetes status, our analysis revealed that retinopathy was significantly associated with greater foot temperature asymmetry. This finding is clinically meaningful, as retinopathy reflects underlying microvascular dysfunction, which may also contribute to impaired circulation and thermal regulation in the feet. This reinforces the concept that diabetic complications are interconnected, and thermography could serve as a noninvasive window into broader microvascular health.

The technology offers a promising approach to identifying early signs of DFUs before these ulcers become visible without specialized tools. This capability implies that it could serve as an effective early warning system, potentially allowing for preventive measures to be taken before the condition worsens and becomes more challenging to treat.

### Comparison With Previous Work

Using technology to predict foot ulcers could play a vital role in the management of diabetes. Several studies have assessed the effectiveness of thermology in detecting abnormal temperature patterns among patients with mild diabetes [19,20,28]. It has been shown that the peripheral vessels and nerves are damaged producing an irregular thermoregulation of both feet [23,36]. However, there is a lack of prospective studies that used AI-powered thermography technology to detect these abnormal patterns.

Some of our findings confirm those previously reported in the context of using a human thermographer to detect DFUs. Our findings are in agreement with those of Hernandez-Contreras et al [20], Ilo et al [19], and Schaper et al [28], who concluded that thermography revealed local temperature differences in high-risk diabetic feet. However, these studies relied on a human thermographer to do the analysis while we leveraged an automated AI-powered software. It is the novel part of this study.

### Strengths and Limitations

The RA who was collecting the data was blinded to the results of the technology. An independent analyst compared the data between the group with diabetes and the healthy group. The use of AI and portable thermal cameras could enhance access to thermology and diabetic foot screening. It could also improve access throughout sparsely populated rural areas as they can access information remotely.

A limitation of this study is that the population with diabetes was not specifically stratified based on the duration of diabetes or the presence or absence of PAD. However, we did include self-reported data on PAD to mitigate its potential impact. While this approach might not be as robust as clinical verification, it helps ensure that any effect on the thermal imaging results is minimal. Although attempts were made to diversify recruitment, the findings should be interpreted with caution regarding their applicability to other racial or ethnic populations. Thus, studies with different ethnic populations should be performed. Another limitation of this study is that it was clinic-based and hence there could have been some referral bias in the selection of the participants. Finally, the sample size was relatively small and we lack a formal sample size calculation. In future research, we plan to perform formal sample size estimations based on preliminary data or expected effect sizes to enhance the statistical power and reliability of our studies. Nevertheless, despite these constraints, the outcomes imply that thermal imaging could serve as a beneficial supplementary resource within primary health care clinics.

### Implications for Practice and Future Research

**Figure 3.**
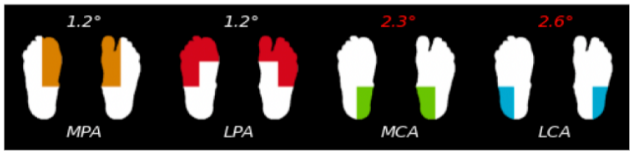
Asymmetry divided by angiosomes. LCA: lateral calcaneal artery; LPA: lateral plantar artery; MCA: medial calcaneal artery; MPA: medial plantar artery.

The study aims to provide evidence of differences in plantar thermal patterns detected by computer vision between adult patients with diabetes with no visible foot ulcers and healthy individuals with no diabetes. The use of thermography is increasingly gaining importance in the early detection of DFUs [18-20]. Hernandez-Contreras et al [20] highlighted how thermography can adequately pinpoint local hotspots in patients with diabetes, aiding in uncovering subclinical infections and discovering areas of high plantar pressure, where early identification is key to effective management. Furthermore, Schaper et al [28] found that thermography could provide early clinical insights before visible signs of foot ulcers. We have included a temperature analysis image (Figure 3) to illustrate how the system identifies areas of concern, including the angiosome divisions. This detailed representation allows individuals with diabetes and their health care providers to focus on specific regions of the foot for targeted care and monitoring. Several researchers recommend regular thermogram assessments for patients with diabetes, even in those with controlled diabetes, since high glucose levels can damage blood vessels and nerves at any time [23,28,36].

Early active intervention can significantly lower the incidence of foot ulcers and amputations in people with diabetes [37]. Therefore, it is essential to diagnose and treat DFUs early. An annual foot examination for people with diabetes is recommended to find high-risk conditions. Depending upon findings, more frequent assessments may be required, as recommended by the International Working Group on the Diabetic Foot [29]. Patients should receive professional diabetic foot care if they have 1 or more high-risk foot conditions [3]. Many pharmacological and nonpharmacological interventions are available to promote blood circulation in diabetic feet.

This study provides insights into the effectiveness of the technology in identifying early signs of DFUs. The key advantage of the technology is that it leverages core thermography principles while eliminating the need for a specialized thermographer. This attribute significantly enhances its use, enabling a broader range of health care practitioners to use this technology. The system could automatically generate a report of the findings and share it with the health care providers or the patient. If thermography shows a compromised blood supply to a specific angiosome in patients with diabetes, we can focus more on preventing the development of diabetic ulcers in that area. Health care providers can view the data to determine whether additional testing or procedures are necessary to avoid foot complications or amputations. If an intervention is carried out at an early stage, it is expected that serious foot complications can be prevented and treated [17,19,20]. The integration of the technology into clinical practice has the potential to offer a more accessible, efficient, and effective approach to managing the risks associated with DFUs.

We are conducting additional studies to compare AI-based thermography with the assessment of a health care professional. In addition, future studies should examine skin temperature maps and how they correspond with patient symptoms, conditions, and disease stages. A larger and more heterogeneous sample with a longer follow-up period could confirm the study findings and expand the knowledge around the effectiveness of the technology in predicting DFUs. In addition, future studies should evaluate the feasibility of the technology as a complementary diagnostic tool or screening test for DFUs. It is important to conduct further studies to better understand the relationship between the unusual heat signatures that were detected and the actual development of DFUs. Understanding this connection could significantly enhance our ability to predict and prevent these ulcers, improving patient outcomes and reducing the need for more invasive treatments.

### Conclusions

DFU significantly impacts the morbidity and mortality of patients with diabetes, with early detection being crucial in limiting its progression and the potential for amputation. This study introduces the use of AI as an effective tool for early detection. It validated a system that detects plantar thermal patterns, distinguishing between healthy individuals and patients with diabetes with no visible ulcers, to demonstrate its potential in identifying early diabetic foot complications. Our findings indicate that the technology can distinguish between the thermal patterns of patients with diabetes and healthy individuals, highlighting its capability to enhance early diagnosis and outcomes in diabetic foot care. It can identify compromised blood supply in patients with diabetes, which suggests that it could play a crucial role in targeted prevention strategies in clinical practice.

## Abbreviations

AAT: American Academy of Thermology
AI: artificial intelligence
DFU: diabetic foot ulcer
HbA_1c_: hemoglobin A_1c_
OLS: ordinary least squares
PAD: peripheral artery disease
RA: research assistant

## References

[1] Sun H, Saeedi P, Karuranga S (2023). Erratum to “IDF Diabetes Atlas: global, regional and country-level diabetes prevalence estimates for 2021 and projections for 2045” [Diabetes Res. Clin. Pract. 183 (2022) 109119]. Diabetes Res Clin Pract.

[2] Namazi N, Moghaddam SS, Esmaeili S (2024). Burden of type 2 diabetes mellitus and its risk factors in North Africa and the Middle East, 1990-2019: findings from the Global Burden of Disease study 2019. BMC Public Health.

[3] van Netten JJ, van Baal JG, Liu C, van der Heijden F, Bus SA (2013). Infrared thermal imaging for automated detection of diabetic foot complications. J Diabetes Sci Technol.

[4] Edmonds M, Manu C, Vas P (2021). The current burden of diabetic foot disease. J Clin Orthop Trauma.

[5] Armstrong DG, Swerdlow MA, Armstrong AA, Conte MS, Padula WV, Bus SA (2020). Five year mortality and direct costs of care for people with diabetic foot complications are comparable to cancer. J Foot Ankle Res.

[6] Driver VR, Fabbi M, Lavery LA, Gibbons G (2010). The costs of diabetic foot: the economic case for the limb salvage team. J Am Podiatr Med Assoc.

[7] Kurkela O, Lahtela J, Arffman M, Forma L (2023). Infrared thermography compared to standard care in the prevention and care of diabetic foot: a cost analysis utilizing real-world data and an expert panel. Clinicoecon Outcomes Res.

[8] Everett E, Mathioudakis N (2018). Update on management of diabetic foot ulcers. Ann N Y Acad Sci.

[9] Miranda C, Da Ros R, Marfella R (2021). Update on prevention of diabetic foot ulcer. Arch Med Sci Atheroscler Dis.

[10] Campbell JS, Mead MN (2022). Human Medical Thermography.

[11] Serbu G (2009). Infrared imaging of the diabetic foot. InfraMation Proc.

[12] Lahiri BB, Bagavathiappan S, Jayakumar T, Philip J (2012). Medical applications of infrared thermography: a review. Infrared Phys Technol.

[13] Zakaria SA, Low CL, Kow RY (2024). Thermography research in diabetic foot: insights from a Scopus-based bibliometric study. Cureus.

[14] Schwartz RG, Brioschi ML, O’Young B (2021). The American Academy of Thermology Guidelines for Neuro-Musculoskeletal 2021: Infrared Medical Thermology & Sympathetic Skin Response (SSR) Studies. Pan Am J Med Thermol.

[15] Ilo A (2020). Infrared Thermography in Vascular Disorders: Screening and Follow-Up.

[16] Nagase T, Sanada H, Takehara K (2011). Variations of plantar thermographic patterns in normal controls and non-ulcer diabetic patients: novel classification using angiosome concept. J Plast Reconstr Aesthet Surg.

[17] de Deus Passos M, da Rocha AF (2022). Evaluation of infrared thermography with a portable camera as a diagnostic tool for peripheral arterial disease of the lower limbs compared with color Doppler ultrasonography. Arch Med Sci Atheroscler Dis.

[18] Zhou Q, Qian Z, Wu J, Liu J, Ren L, Ren L (2021). Early diagnosis of diabetic peripheral neuropathy based on infrared thermal imaging technology. Diabetes Metab Res Rev.

[19] Ilo A, Romsi P, Mäkelä J (2020). Infrared thermography and vascular disorders in diabetic feet. J Diabetes Sci Technol.

[20] Hernandez-Contreras DA, Peregrina-Barreto H, Rangel-Magdaleno J de J, Renero-Carrillo FJ (2019). Plantar Thermogram Database for the Study of Diabetic Foot Complications. IEEE Access.

[21] Sudha BG, Umadevi V, Shivaram JM, Sikkandar MY, Al Amoudi A, Chaluvanarayana HC Statistical analysis of surface temperature distribution pattern in plantar foot of healthy and diabetic subjects using thermography.

[22] Bagavathiappan S, Philip J, Jayakumar T (2010). Correlation between plantar foot temperature and diabetic neuropathy: a case study by using an infrared thermal imaging technique. J Diabetes Sci Technol.

[23] Papanas N, Papatheodorou K, Papazoglou D, Kotsiou S, Maltezos E (2010). Association between foot temperature and sudomotor dysfunction in type 2 diabetes. J Diabetes Sci Technol.

[24] Araújo A de, Negreiros F da S, Florêncio RS, Oliveira S de, Silva A da, Moreira TMM (2022). Effect of thermometry on the prevention of diabetic foot ulcers: a systematic review with meta-analysis. Rev Lat Am Enfermagem.

[25] Ena J, Carretero-Gomez J, Arevalo-Lorido JC, Sanchez-Ardila C, Zapatero-Gaviria A, Gómez-Huelgas R (2021). The association between elevated foot skin temperature and the incidence of diabetic foot ulcers: a meta-analysis. Int J Low Extrem Wounds.

[26] Houghton VJ, Bower VM, Chant DC (2013). Is an increase in skin temperature predictive of neuropathic foot ulceration in people with diabetes? A systematic review and meta-analysis. J Foot Ankle Res.

[27] Armstrong DG, Holtz-Neiderer K, Wendel C, Mohler MJ, Kimbriel HR, Lavery LA (2007). Skin temperature monitoring reduces the risk for diabetic foot ulceration in high-risk patients. Am J Med.

[28] Schaper NC, van Netten JJ, Apelqvist J (2024). Practical guidelines on the prevention and management of diabetes‐related foot disease (IWGDF 2023 update). Diabetes Metabolism Res.

[29] Basatneh R, Najafi B, Armstrong DG (2018). Health sensors, smart home devices, and the internet of medical things: an opportunity for dramatic improvement in care for the lower extremity complications of diabetes. J Diabetes Sci Technol.

[30] Fraiwan L, AlKhodari M, Ninan J, Mustafa B, Saleh A, Ghazal M (2017). Diabetic foot ulcer mobile detection system using smart phone thermal camera: a feasibility study. Biomed Eng Online.

[31] Vilcahuaman L, Harba R, Canals R Automatic analysis of plantar foot thermal images in at-risk type II diabetes by using an infrared camera.

[32] Kanazawa T, Nakagami G, Goto T (2016). Use of smartphone attached mobile thermography assessing subclinical inflammation: a pilot study. J Wound Care.

[33] Qin Q, Nakagami G, Ohashi Y, Dai M, Sanada H, Oe M (2022). Development of a self-monitoring tool for diabetic foot prevention using smartphone-based thermography: plantar thermal pattern changes and usability in the home environment. Drug Discov Ther.

[34] Murphy CA, Laforet K, Da Rosa P, Tabamo F, Woodbury MG (2012). Reliability and predictive validity of Inlow’s 60-second diabetic foot screen tool. Adv Skin Wound Care.

[35] Renero-C FJ (2017). The thermoregulation of healthy individuals, overweight-obese, and diabetic from the plantar skin thermogram: a clue to predict the diabetic foot. Diabet Foot Ankle.

[36] Chatwin KE, Abbott CA, Boulton AJM, Bowling FL, Reeves ND (2020). The role of foot pressure measurement in the prediction and prevention of diabetic foot ulceration—a comprehensive review. Diabetes Metab Res Rev.

[37] Lung CW, Wu FL, Liao F, Pu F, Fan Y, Jan YK (2020). Emerging technologies for the prevention and management of diabetic foot ulcers. J Tissue Viability.

